# Anti-phospholipase A2 Receptor Antibody-Negative Membranous Nephropathy in Pregnancy

**DOI:** 10.7759/cureus.42827

**Published:** 2023-08-01

**Authors:** Maryam Saleem, Hassaan Iftikhar

**Affiliations:** 1 Nephrology, Ohio Valley Nephrology Associates, Owensboro, USA; 2 Nephrology, Washington University School of Medicine, St. Louis, USA; 3 Internal Medicine, Waterbury Hospital, Waterbury, USA; 4 Internal Medicine, Saint Francis Medical Center, Trenton, USA

**Keywords:** nephrotic-range proteinuria, pla2r, pregnancy, nephrotic syndrome, membranous nephropathy

## Abstract

Nephrotic syndrome in pregnancy is associated with challenges for both patients and physicians. Early recognition is crucial, and when appropriate, renal biopsy should be considered to differentiate preeclampsia from other causes of glomerulopathies. Pregnancy in a woman with nephrotic syndrome is high risk, and more data are needed to highlight pregnancy outcomes.

## Introduction

Nephrotic syndrome is a rare occurrence in pregnancy. Most cases involve previously diagnosed glomerulopathies or preeclampsia, while new-onset primary glomerulopathies rarely occur. Here, we report a case of phospholipase A2 receptor antibody (PLA2R)-negative membranous nephropathy in a pregnant female with no prior history of proteinuria.

## Case presentation

A 19-year-old Hispanic female, G1P0, 21 weeks pregnant, presented to the hospital with lower extremity swelling for about four weeks, associated with 14 pounds weight gain. This was the patient's first pregnancy, and it had been uneventful until this presentation. Four weeks prior to the current presentation, the patient presented to an emergency room (ER) for a urinary tract infection and was treated with nitrofurantoin, resulting in the resolution of the infection, but she developed progressive edema of the lower extremities. Review of systems was positive for fatigue and foamy urine. The physical exam was consistent with diffuse lower extremity and groin edema. Her blood pressure on presentation was 116/68 mmHg and stayed at <120/80 mmHg throughout the hospital stay. Laboratory workup revealed serum creatinine of 0.7 mg/dL (baseline 0.7 mg/dL about a month ago, reference range: 0.7-1.3 mg/dL), albumin of 1.3 g/dL (reference range: 3.5-5.7 g/dL), hemoglobin of 12.5 g/dl (reference range: 12.1-15.1 g/dL), platelet count of 175,000/uL (reference range: 150,000-400,000/uL), and lactate dehydrogenase of 200 units/L (reference range: 105-333 units/L). Urinalysis showed 3+ protein and absence of hematuria, and 24-hour urine studies confirmed 8 grams of proteinuria. Urinalysis in the first trimester did not show any proteinuria or hematuria. Additional workup was obtained to determine the etiology of underlying nephrotic syndrome, as shown in Table 1. 

**Table 1:** Serologic workup performed prior to biopsy IgM: immunoglobulin M

Test Type	Result
Human immunodeficiency virus	Non-reactive
Hepatitis A IgM	Non-reactive
Hepatitis B surface antigen and core IgM	Non-reactive
Hepatitis C antibody	Non-reactive
Rapid plasma reagin	Non-reactive
Antinuclear antibody	Not detected
Anti-double stranded DNA	Not detected
Antineutrophil cytoplasmic antibody	Not detected
Complement (C3 and C4)	Normal
Anti-glomerular basement membrane antibody	Not detected
Phospholipase A2 receptor (PLA2R) antibody	Not detected

**Figure 1:**
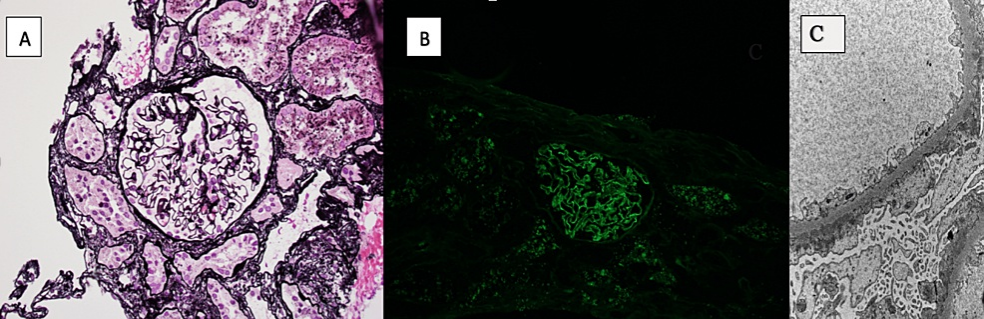
(A) Light microscopy silver stain, (B) IgG immunofluorescence stain, (C) electron microscopy image (A) Light microscopy silver stain revealing diffuse thickening of the glomerular basement membrane; (B) immunofluorescence microscopy revealing a diffuse granular pattern of IgG; (C) electron microscopy revealing subepithelial electron-dense deposits on the glomerular basement membrane and effacement of the podocyte foot processes

Renal biopsy was performed during the same hospitalization during the second trimester, week 22, which showed membranous nephropathy, as shown in Figure 1A and Figure 1B. Immunofluorescence (IF) for PLA2R staining was negative. There was minimal interstitial fibrosis.

The patient was treated with prednisone 1 mg/kg (70 mg) and tacrolimus (1 mg twice a day with goal range of 3-7 ng/ml) and received diuresis with furosemide. Renal function did not decline, and edema improved significantly. Proteinuria was reduced to 6 gram on repeat 24-hour urine collection, and serum albumin improved to 1.9 g/dl after one week of treatment during the same hospital stay. Through the hospital stay, fetal development was also monitored by the obstetrics department, and no abnormalities were seen. The patient was also started on enoxaparin as she was at high risk for hypercoagulopathy due to pregnancy with severe nephrotic syndrome. The patient was discharged home, but was unfortunately lost to follow-up afterwards.

## Discussion

Although primary membranous nephropathy (pMN) remains one of the common causes of nephrotic syndrome in adults, very limited data are available about its incidence in pregnant patients [1]. Most of the data are obtained from case reports or case series regarding maternal-fetal outcomes in pregnant patients with nephrotic syndrome [2]. While some of these cases of pMN are diagnosed before pregnancy [3-5], there have been cases of pMN first diagnosed during pregnancy when patients presented with nephrotic syndrome [6-8]. Interestingly, only in very few case reports, the serum PLA2R antibody level was checked [6-7]. PLA2R is an important target in the pathogenesis of pMN and is associated with poor outcomes in pregnancy [9]. pMN from antigens other than PLA2R in pregnancy has not been described in the literature. pMN (PLA2R positive or negative) is usually diagnosed in the first or second trimester of pregnancy and presents as hypoalbuminemia and nephrotic-range proteinuria although maternal renal function may decline during pregnancy. The outcomes of pregnancy vary, with a literature review suggesting reduced impact of pMN on fetal outcomes [10]. However, more recently, pMN, just like other nephrotic syndromes, increases the risk of unfavorable fetal outcomes, such as stillbirth, premature delivery, or intrauterine growth restriction [9,11]. The treatment of pMN in pregnancy includes management of volume overload with loop diuretics. Both nephrotic syndrome and pregnancy are associated with hypercoagulability, so anticoagulation should be considered especially when there is profound hypoalbuminemia [12]. The choice of immunosuppression is very limited and includes steroids and calcineurin inhibitors (CNIs) [3-5] although rituximab has also been used [5]. 

Given that the literature is devoid of disease-specific data for primary glomerular diseases, clinicians must assess the cases of proteinuria in pregnancy very carefully, utilizing a multidisciplinary team (including nephrologists, obstetricians, and maternal fetal medicine specialists) approach for care of these patients. Because of the complexity and ethical concerns of these high-risk pregnancies and pregnancy generally being an exclusion criteria for most clinical trials, conducting a randomized controlled trial to assess the course of kidney disease, treatment responses, and pregnancy outcomes can be very challenging. However, case reports and retrospective case series are critical in guiding medical decision-making.

## Conclusions

Our case is unique as the patient presented with nephrotic syndrome in her second trimester (the literature suggests that patients usually present in their first trimester) and had PLA2R-negative membranous nephropathy (pMN in pregnancy from antigens other than PLA2R has not been described in the literature). Whether pMN will affect the patient’s subsequent pregnancies or not remains unknown, making it extremely difficult for counseling for future pregnancies in such a patient population. The clinicians must assess cases of proteinuria in excess of 300 mg in pregnancy carefully and ideally obtain renal biopsy if possible and safe to do so for diagnostic clarification and for improvement of maternal and fetal outcomes.
